# Preliminary *in vitro* and *in vivo* evaluation of liposomal nanoparticles for passive and active tumour targeting by scintigraphic and MRI imaging

**DOI:** 10.1186/2197-7364-1-S1-A82

**Published:** 2014-07-29

**Authors:** Bianchi Patrick, Fragogeorgi Eirini, Efthymiadou Eleni, Xanthopoulos Stavros, Psimadas Dimitrios, Bouzioti Penelope, Kordas George, Loudos George, Silvio Aime

**Affiliations:** Department of Molecular Biotechnology and Health Sciences, Molecular Imaging Center, University of Torino, Torino, Italy; Department of Biomedical Engineering, Technological Educational Institute of Athens, Athens, Greece; NCSR Democritos, Institute of Nuclear & Radiological Sciences & Technology, Energy & Safety, Aghia Paraskevi, Greece; NCSR Democritos, Institute of Advanced Materials, Physicochemical processes, Nanotechnology & Microsystems, Athens, Greece

Liposomes belong to the most desirable drug delivery systems that enable their application in MRI and SPECT by incorporating lipids chelated to MR contrast agents as well as to radioactive tracers. Here we report our observation on a novel αvβ3- targeting liposome formulation (RGD-LP) and on the corresponding non-targeting control (NT-LP) for diagnosis and molecular imaging of tumor by exploiting SPECT/MRI technology.

Radiolabelling was performed with ^99m^Tc tricarbonyl core via the ligand cysteine (Cys). Both peptide and ligand were coupled to DSPE-PEG_2000_-Mal lipid. Radiochemical purity and *in vitro* stability tested in the presence of competitive ligands for ^99m^Tc^(I)^ were assessed by paper chromatography. For nuclear imaging a custom SPECT system (1.5mm spatial resolution) was used and the results were correlated to the biodistribution data. Both liposomes disposed of a PEG surface density of 5.5% and were loaded with 25% of Gd-based lipid (B2286) for MRI studies. For recording MR images, cells were transferred into glass capillaries, centrifuged and placed in an agar phantom. MR images were acquired on a Bruker Avance300 spectrometer operating at 7.1 T. *In vivo* MRI experiments were carried out on U87MG tumor xenografts with a 1T scanner (Bruker, Icon Instrument).

Radiolabelling yield (>80%), radiochemical purity (>90%), *in vitro stability* in the presence of excess amount of cysteine and in 10% FBS/DMEM were satisfactory to permit *in vivo* evaluation at normal mice. NT-LP-Cys showed higher blood retention and lower uptake at liver and spleen at later time points than RGD-LP which is in good correlation with the *in vivo* MRI results obtained in U87MG bearing mice. The internalization amount of Gd^3+^ inside the cells was of higher extent for RGD-LP at all the time points analyzed.

These results could be considered as an important step in the development of tumour targeted SPECT/MRI contrast agents for cancer imaging.

